# Maternal Cardiac Arrest During Cesarean Section in the Setting of Severe Preeclampsia and Uncontrolled Type 1 Diabetes: A Case Report

**DOI:** 10.7759/cureus.102348

**Published:** 2026-01-26

**Authors:** Keren Khromchenko, Deborah Winograd, Jonathan Faro, Paulina Sedutto

**Affiliations:** 1 Obstetrics and Gynecology, Hackensack Meridian Jersey Shore University Medical Center, Neptune, USA; 2 Maternal-Fetal Medicine, Hackensack Meridian Jersey Shore University Medical Center, Neptune, USA

**Keywords:** cardiovascular risk (cvr), maternal cardiac arrest, obstetric case report, severe preeclampsia, type 1 diabetes mellitus

## Abstract

Maternal cardiac arrest is an uncommon but life-threatening complication of pregnancy. Several maternal, social, and obstetric factors have been associated with increased risk, including older maternal age, underlying medical comorbidities, and hypertensive disorders of pregnancy. We present a case of maternal cardiac arrest during cesarean section in a patient with preeclampsia with severe features and uncontrolled type 1 diabetes. This case raises awareness for maternal morbidity and suggests cardiovascular risk and preconception counseling in high-risk obstetrical patients.

A 31-year-old G2P1001 at 32 weeks and 1 day of gestation presented with preeclampsia with severe features and uncontrolled type 1 diabetes. On hospital day 2, the patient developed pulmonary edema. Because of this, magnesium sulfate for seizure prophylaxis was discontinued, and delivery via repeat cesarean section was performed. During surgery, the patient became agitated, which persisted despite sedation. She had an episode of oxygen desaturation followed by bradycardia to 36 beats per minute. The patient was intubated, and asystole was recognized. Advanced cardiac life support was initiated, and the patient was resuscitated. The bedside echocardiogram showed an ejection fraction of 25-30%. She was diagnosed with cardiogenic shock and treated with vasopressors and insertion of an Impella device. The patient’s condition was most likely exacerbated by her morbidities: preeclampsia with severe features and uncontrolled diabetes.

Preeclampsia with severe features and diabetes can be considered as independent risk factors for maternal cardiac arrest. Diabetes and hypertension are known risk factors for heart disease, which can be amplified in the setting of physiologic changes that occur during pregnancy. This case demonstrates the role of cardiometabolic disease in peripartum cardiovascular collapse and emphasizes the importance of cardiovascular risk stratification, preconception counseling, and multidisciplinary surveillance in high-risk patients.

## Introduction

Maternal cardiac arrest is a rare but serious complication of pregnancy. Although hemorrhage is a common cause, cardiometabolic factors, including smoking, diabetes, and hypertension, also play an important role [1]. With many women delaying childbearing, heart disease has emerged as a major contributor to maternal mortality, accounting for a quarter of maternal deaths in the United States between 2006 and 2010 [2]. Conditions such as diabetes and hypertensive disorders of pregnancy are increasingly recognized as important risk factors for maternal cardiovascular complications. Hypertensive disorders of pregnancy are a leading cause of maternal and perinatal mortality worldwide and include chronic hypertension (diagnosed before 20 weeks’ gestation), gestational hypertension (diagnosed after 20 weeks’ gestation), and preeclampsia with or without severe features, characterized by hypertension with proteinuria and/or end-organ dysfunction [3]. Recognition of cardiometabolic risk factors prior to conception offers an opportunity for risk stratification and pre-pregnancy counseling aimed at reducing adverse maternal and fetal outcomes.

Our case report illustrates the interplay of cardiometabolic disease and hypertensive disorders of pregnancy in the development of acute maternal cardiac morbidity. We describe a case of maternal cardiac arrest during cesarean delivery, precipitated by heart failure. This patient’s risk factors include uncontrolled type 1 diabetes, preeclampsia with severe features, prior history of gestational hypertension, and exposure to spinal anesthesia. This case highlights the importance of pre-pregnancy counseling and close surveillance during pregnancy in optimizing women’s health to reduce adverse maternal and fetal outcomes in the peripartum period.

## Case presentation

Our patient is a 31-year-old G2P1001 with a history of poorly controlled type 1 diabetes, gestational hypertension, one prior cesarean section, and a body mass index (BMI) of 31 kilograms/meters^2^. She presented at 32 weeks and 1 day of gestation due to hypertension and nausea. Physical examination was normal, with lungs clear to auscultation bilaterally. 

In triage, the patient’s blood pressures were 194/121 mmHg and 185/106 mmHg, recorded 15 minutes apart. Oxygen saturation was 96% on room air, and her heart rate was 116 beats per minute. She was promptly administered labetalol 20 mg IV, after which her blood pressure was within a mild range. Urine protein-to-creatinine ratio was 12,040 mg/g and blood sugar measured 242 mg/dL, while other labs were unremarkable (Table 1). Due to persistent severe-range blood pressures requiring intravenous antihypertensive medications, the patient was diagnosed with preeclampsia with severe features. 

**Table 1:** Laboratory Results on Admission Key abnormalities: severe proteinuria and hyperglycemia. Abnormal laboratory values are bolded. µL: microliter; g: grams; dL: deciliter; mg: milligrams; U: units; L: liter.

Lab Type	Patient’s Result	Reference Value
White blood cells (×10^3^/µL)	11.2	4.5-11
Hemoglobin (g/dL)	13.7	12-16
Hematocrit (%)	41.1	35.0-48.0
Platelets (×10^3^/µL)	495	140-450
Glucose (mg/dL)	242	70-99
Creatinine (mg/dL)	0.72	0.44-1.00
Aspartate aminotransferase (U/L)	21	10-42
Alanine aminotransferase (U/L)	12	10-60
Urine protein/creatinine ratio (mg/g)	12,040.0	<200.0
Lactate dehydrogenase (U/L)	243	91-200
Uric acid (mg/dL)	5.2	4.0-8.0

The patient was admitted and started on magnesium sulfate for seizure prophylaxis, betamethasone for fetal lung maturity, labetalol for blood pressure control, and insulin for glycemic management. On hospital day 2, early morning labs, including creatinine level, and aspartate aminotransferase and alanine aminotransferase levels, remained within normal limits (Table 2). However, about 4 hours later, the patient's oxygen saturation began trending persistently below 95%. Pulmonary exam revealed inspiratory crackles in the right lower lobe. Given the clinical concern for pulmonary edema in the context of severe preeclampsia, expectant management was deemed inappropriate, and magnesium was discontinued. The decision was made to proceed with delivery via repeat cesarean section.

**Table 2:** Morning Laboratory Results on Hospital Day 2 Key abnormality: hyperglycemia. Abnormal laboratory values are bolded. µL: microliter; g: grams; dL: deciliter; mg: milligrams; U: units; L: liter.

Lab Type	Patient’s Result	Reference Value
White blood cells (×10^3^/µL)	9.0	4.5-11
Hemoglobin (g/dL)	12.2	12-16
Hematocrit (%)	36.1	35.0-48.0
Platelets (×10^3^/µL)	437	140-450
Glucose (mg/dL)	144	70-99
Creatinine (mg/dL)	0.63	0.44-1.00
Aspartate aminotransferase (U/L)	19	10-42
Alanine aminotransferase (U/L)	10	10-60
Lactate dehydrogenase (U/L)	210	91-200
Uric acid (mg/dL)	5.6	4.0-8.0

The cesarean section was performed under spinal anesthesia. Estimated blood loss was 308 mL. The infant was delivered atraumatically, weighing 2670 grams with APGAR scores of 6 and 8 at 1 and 5 minutes, respectively. The whole procedure lasted approximately 50 minutes, with rupture of membranes taking place 18 minutes after skin incision and fetal and placental delivery occurring 1 minute later.

**Figure 1:**
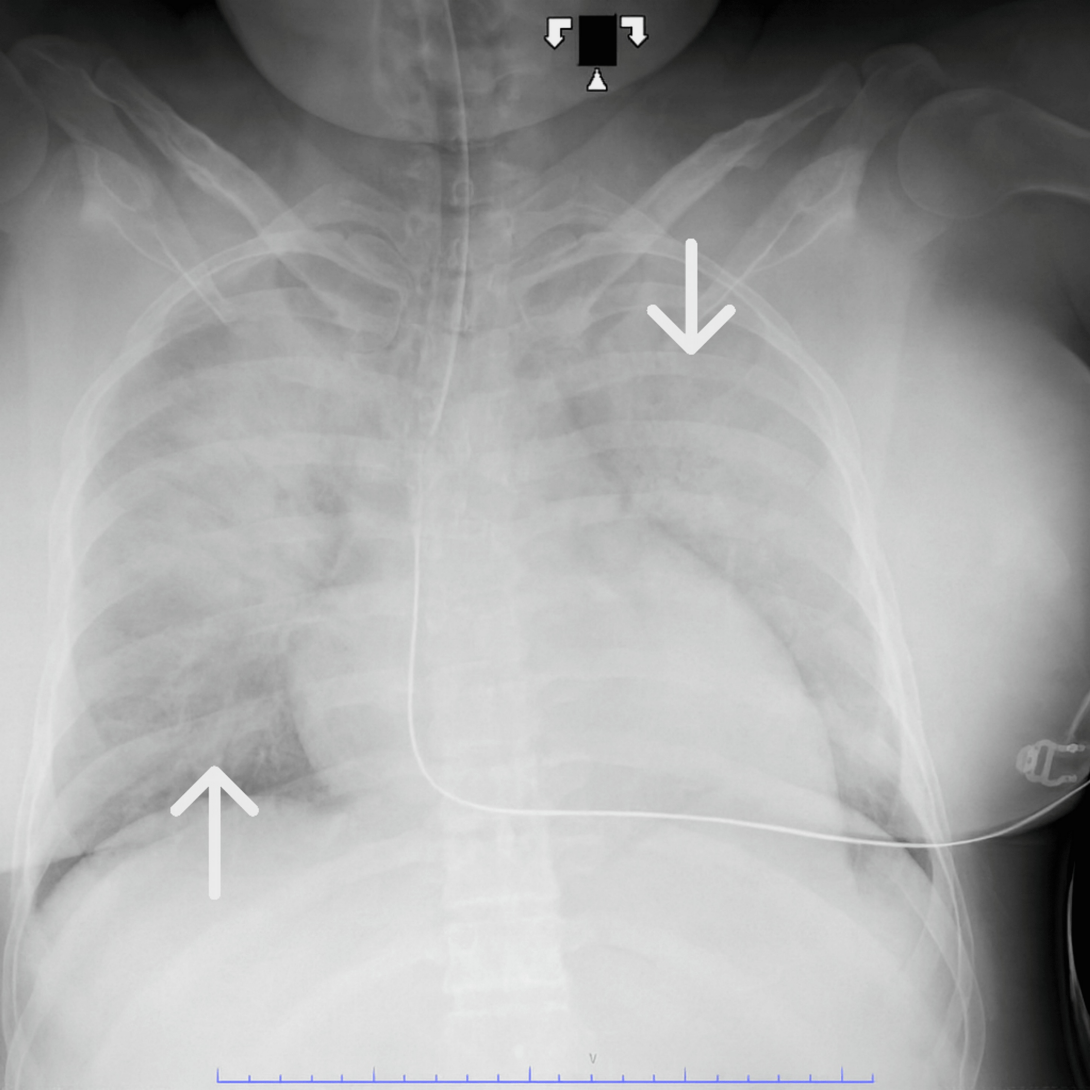
Patient's Chest X-Ray Demonstrating Pulmonary Edema Bilaterally The white arrows are pointing to bilateral consolidations with air bronchograms consistent with pulmonary edema.

**Figure 2:**
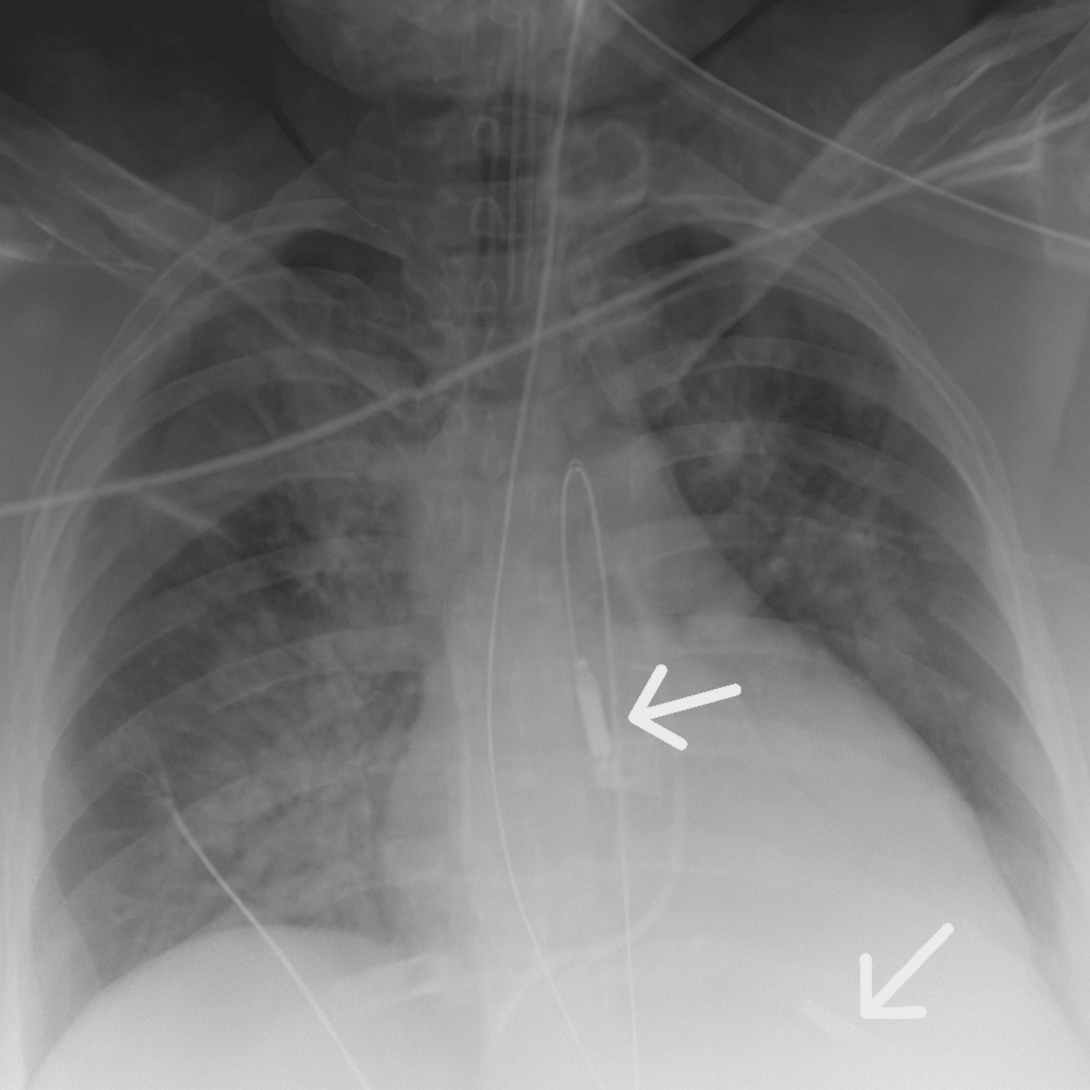
Chest X-Ray With Impella Device White arrows are demonstrating the Impella device in place.

**Figure 3:**
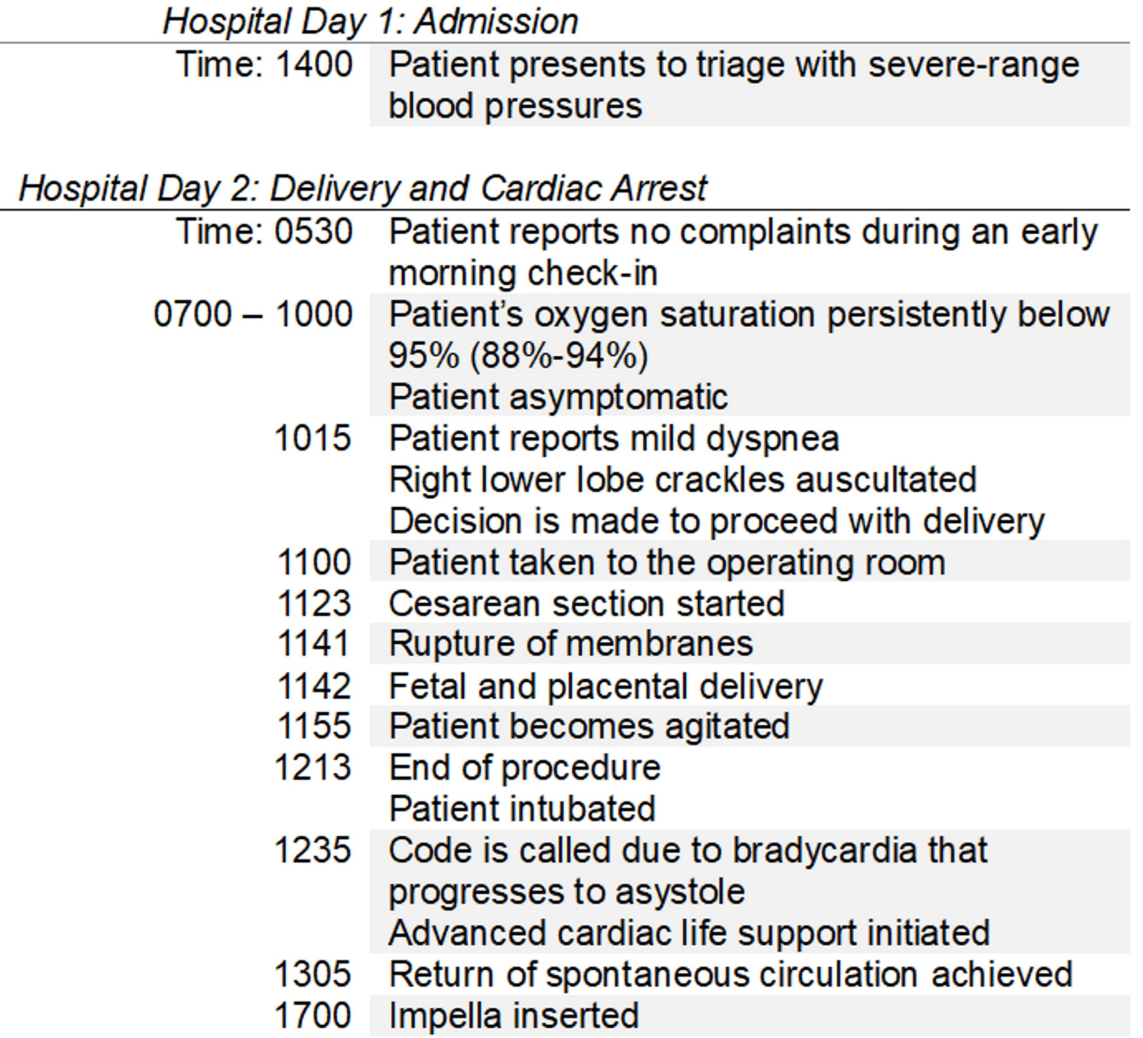
Timeline of Key Events Leading Up to Cardiac Arrest The left-hand side of the figure lists the time an event took place, and the right-hand side lists the key event.

During closure of the rectus muscle, approximately 13 minutes after placental delivery, the patient became increasingly agitated and poorly responsive to sedation. At that time, vital signs demonstrated hypotension with a blood pressure of 88/54 mmHg, a heart rate of 93 beats per minute, a respiratory rate of 32 breaths per minute, and an oxygen saturation of 95%. Immediately following skin closure, she developed worsening hypoxia with oxygen saturation declining to 89%, necessitating endotracheal intubation. A code was called 22 minutes after intubation as a result of profound bradycardia to a rate of 36 beats per minute, which progressed to asystole. Advanced cardiac life support was initiated, and return of spontaneous circulation was achieved after 30 minutes of resuscitation. A bedside echocardiogram demonstrated an estimated ejection fraction of 25-30% with severe global hypokinesis. Chest X-ray confirmed pulmonary edema (Figure 1). Due to these clinical findings, the interventional cardiology team inserted an Impella device to improve hemodynamic support (Figure 2). The patient was then transferred to the intensive care unit. Labs obtained immediately after delivery and 6 hours after demonstrated worsening kidney and liver function, supporting the patient's acute clinical deterioration secondary to preeclampsia with severe features (Table 3). Figure 3 is a timeline of key events that took place leading up to the patient's cardiac arrest. 

**Table 3:** Laboratory Results Immediately and 6 Hours After Delivery Key abnormalities: hyperglycemia and worsening kidney and liver function. Abnormal laboratory values are bolded. *In the setting of normal fibrinogen and prothrombin time, partial thromboplastin time is likely elevated due to patient's receipt of a heparin injection prior to lab draw. Heparin was initiated after insertion of the Impella device for anticoagulation prophylaxis. µL: microliter; g: grams; dL: deciliter; mg: milligrams; U: units; L: liter.

Lab Type	Immediately After	Six Hours After	Reference Value
White blood cells (×10^3^/µL)	22.3	21.3	4.5-11
Hemoglobin (g/dL)	12.3	9.8	12-16
Hematocrit (%)	37.4	29.9	35.0-48.0
Platelets (×10^3^/µL)	454	351	140-450
Glucose (mg/dL)	334	409	70-99
Creatinine (mg/dL)	1.04	1.12	0.44-1.00
Aspartate aminotransferase (U/L)	584	553	10-42
Alanine aminotransferase (U/L)	213	166	10-60
Fibrinogen (mg/dL)	511	255	232-519
Prothrombin time (seconds)	10.1	12.5	10.2-13.5
Partial thromboplastin time (seconds)	35	197*	26-39

On postoperative day 2, the patient was extubated, weaned off of vasopressors, and the Impella device was explanted. Her remaining hospital course was complicated by persistent hypertension requiring medication adjustments, acute kidney injury, and postpartum depression. She was medically stabilized and discharged one week after the cardiac arrest took place. Currently, the patient continues to follow up with her endocrinologist for optimal diabetes control and is clinically doing well.

## Discussion

Pregnancy triggers significant cardiovascular and pulmonary adaptations. Stroke volume, heart rate, and cardiac output increase, while total peripheral resistance decreases [4]. Pulmonary changes include a 20% increase in oxygen consumption and a 5-15% increase in intrapulmonary shunting by the third trimester. Functional residual capacity and thoracic compliance also decline [4]. Additionally, heightened hypercoagulability occurs due to venous stasis, reduced mobility, vascular compression from the gravid uterus, and elevated coagulation factors [5,6]. Increases in stroke volume and estrogen weaken the structural integrity of the aortic wall [5]. These changes make pregnant women less tolerant of oxygen deprivation and at greater risk for airway compromise and catastrophic complications like venous thromboembolism, pulmonary embolism, aortic dissection, acute myocardial infarction, and cardiac arrest [4,5].

As of 2019, the incidence of maternal cardiac arrest is approximately 1/12,000 hospitalizations in the United States [1,4]. The leading cause of this outcome is hemorrhage, followed by anesthetic complications and cardiovascular etiologies [1,4]. Maternal risk factors for cardiac arrest include advanced maternal age, African ancestry, socioeconomic challenges, and comorbidities such as diabetes, pulmonary artery hypertension, cancer, and lupus [1]. Obstetric risk factors for cardiac arrest include fetal death, cesarean delivery, hypertension, placenta previa or accreta, and polyhydramnios [1].

Between 2006 and 2013, cardiovascular disease and cardiomyopathy accounted for 15.5%-26% of pregnancy-related deaths in the United States [2,5]. The diagnosis of heart disease during pregnancy is challenging because symptoms can mimic those experienced in normal pregnancies [2,5]. Our patient suffered from cardiac arrest secondary to heart failure, which was likely exacerbated by preeclampsia with severe features and poorly controlled diabetes. 

Hypertension is one of the most common medical conditions complicating pregnancy, affecting 8-10% of all pregnancies peripartum. It accounts for up to 16% of maternal deaths worldwide [7]. Hypertensive disorders of pregnancy are associated with a two-fold increase in maternal cardiovascular disease risk [8]. Women affected are at an increased short-term and long-term risk for heart failure [7]. A retrospective cohort study found that compared to women with multiple births and no hypertensive disease during pregnancy, women with a history of preeclampsia demonstrated a hazard ratio of 2.00 for the development of heart failure [8]. Another retrospective study observed that compared with normotensive women, there was a progressively increased risk of cardiovascular morbidity during delivery hospitalization with gestational hypertension (adjusted odds ratio (OR), 1.18), preeclampsia without severe features (adjusted OR, 1.96), preeclampsia with severe features (adjusted OR, 3.46), and eclampsia (adjusted OR, 12.46) [9].

Diabetes is an established cardiovascular risk factor, associated with a two- to four-fold increase in the risk of incident heart failure [10,11]. Heart failure may develop due to underlying coronary artery disease, impaired microvascular endothelial function, increased myocardial fibrosis, oxidative stress, and multiple metabolic abnormalities induced by altered glucose metabolism [12]. Glucose transport becomes impaired due to a reduction of cardiac glucose transporters [12]. Energy production in the form of adenosine triphosphate shifts toward beta-oxidation of free fatty acids, a less efficient process that increases oxygen consumption and contributes to cardiac dysfunction during increased metabolic demands or ischemia [12]. Diabetics also exhibit a decrease in sarcolemmal transport and alterations in myofibrillar contractile proteins [12]. Both type 1 and type 2 diabetes may cause structural cardiac changes that can result in heart failure if not treated [13].

While the diagnosis of peripartum cardiomyopathy, defined as systolic cardiac dysfunction in the last month of pregnancy or within five months of delivery in women without pre-existing cardiac disease, was considered, our patient’s pre-existing comorbidities suggest an alternate etiology for heart failure [14]. In addition to gestational hypertension in a prior pregnancy, the patient had worsening preeclampsia with severe features and poorly controlled type 1 diabetes. Both diseases can be associated with significant proteinuria, which contributes to hypovolemia due to decreased plasma oncotic pressure. The resultant reduction in intravascular volume could have predisposed the patient to heart failure.

Another proposed mechanism for the patient’s decompensation is acute afterload stress from uterine autotransfusion. Following the second and third stages of labor, approximately 300-500 mL of blood is shifted back into the maternal circulation, increasing cardiac output by as much as 60-80% [15]. This physiologic process can overwhelm an already compromised cardiovascular system. Notably, our patient began to decompensate after placental delivery, the third stage of labor, coinciding with the timing of autotransfusion.

A rare but serious differential to consider is amniotic fluid embolism. This condition involves the entry of amniotic fluid or fetal debris into maternal circulation, triggering a systemic inflammatory response and activation of procoagulant pathways, often resulting in sudden cardiovascular collapse [16]. This pathology can result in a coagulation disorder called disseminated intravascular coagulation, characterized by the abnormal consumption of coagulation factors and platelets that can lead to life-threatening hemorrhage [17]. A coagulation panel including fibrinogen levels, prothrombin time, and partial thromboplastin time was obtained immediately after and 6 hours after our patient was delivered, as demonstrated in Table 3. Considering these labs had remained stable, and the patient's lochia was appropriate for postpartum, the diagnosis of amniotic fluid embolism was considered less likely. 

Finally, the patient underwent spinal anesthesia, which increases the risk of vasovagal events in pregnant patients due to aortocaval compression syndrome and a higher level of spinal block [18]. The resulting vagal activation and sympathetic blockade can result in significant hypotension, bradycardia, and even cardiac arrest [18].

While the precise cause of the patient’s cardiac arrest remains uncertain, these mechanisms, either alone or in combination, are all plausible contributors.

This case highlights the importance of early identification and prevention of cardiovascular morbidity in high-risk pregnant patients. Pre-pregnancy counseling provides a critical opportunity to optimize patients’ preexisting conditions, including glycemic control and blood pressure management, prior to conception. It also allows patients the opportunity to understand their individualized risks and make informed decisions regarding the timing of pregnancy. During pregnancy, patients with significant risk factors may benefit from cardiovascular risk stratification and multidisciplinary management. This could entail consultations from maternal-fetal medicine physicians, cardiologists, endocrinologists, and anesthesiologists, as well as studies such as electrocardiography or echocardiography. Additionally, educating patients on concerning symptoms such as shortness of breath, chest pain, or intractable headaches can facilitate timely evaluation and intervention. Collectively, these preventive strategies may help mitigate severe maternal cardiovascular complications and improve peripartum outcomes.

## Conclusions

Maternal morbidity and mortality are rising in the United States, driven by increasing maternal age, obesity, diabetes, and hypertension. These cardiometabolic risk factors can contribute to catastrophic peripartum cardiovascular events, such as this case. Early identification of high-risk patients provides an opportunity for pre-pregnancy counseling and cardiovascular risk stratification, in addition to close surveillance throughout pregnancy. Future practice should consider screening echocardiography for high-risk patients, as well as multidisciplinary peripartum planning and surgical optimization, to improve maternal outcomes.
